# Evaluation of the sensitivity of *R*_1_ρ MRI to pH and macromolecular density

**DOI:** 10.1016/j.mri.2019.02.004

**Published:** 2019-05

**Authors:** Syed O. Ali, Petros Fessas, Joshua D. Kaggie, Fulvio Zaccagna, Gavin Houston, Scott Reid, Martin J. Graves, Ferdia A. Gallagher

**Affiliations:** a University of Cambridge School of Clinical Medicine, Addenbrooke's Hospital, Hills Rd, Cambridge CB2 0SP, United Kingdom; b Department of Radiology, Box 218, University of Cambridge, Cambridge CB2 0QQ, United Kingdom; c Cambridge University Hospitals NHS Foundation Trust, Addenbrooke's Hospital, Cambridge, United Kingdom, CB2 0QQ; d GE Healthcare, Amersham, United Kingdom, HP7 9JQ

**Keywords:** MRI, *T*_1_ρ, *R*_1_ρ, pH, Tumor microenvironment

## Abstract

The tumor microenvironment is characteristically acidic and this extracellular acidosis is known to play a role in carcinogenesis and metastasis and can affect tumor chemosensitivity and radiosensitivity. Intracellular pH has been used as a possible biomarker of salvageable tissue in ischemic stroke. A non-invasive MRI-based approach for the determination and imaging of cerebral pH would be a powerful tool in cancer diagnosis and monitoring, as well as stroke treatment planning. Several pH-based MRI imaging approaches have been proposed but for these to be useful, disentangling the effects of pH from other parameters which may affect the measured MRI signal is crucial to ensure accuracy and specificity. *R*_*1*_ relaxation in the rotating frame (*R*_*1*_*_ρ_*) is an example of a method that has been proposed to probe pH *in vivo* using MRI. In this study, we have investigated the relationship between *R*_*1*_*_ρ_*, pH, and macromolecular density *in vitro* using phantoms and in human volunteers. Here we show that the rate of *R*_*1*_*_ρ_* relaxation (=1/*T*_*1*__*ρ*_) varies with pH but only in the presence of macromolecules. At constant pH, phantom macromolecular density inversely correlated with *R*_*1*_*_ρ_*. *R*_*1*_*_ρ_* imaging of the normal human brain demonstrated regional heterogeneity with significant differences between structurally distinct regions, which are likely to be independent of pH. For example, *R*_*1*_*_ρ_* was higher in the basal ganglia compared to grey matter and higher in grey matter compared to white matter. We conclude that *R*_*1*_*_ρ_* cannot be reliably used to image tissue pH without deconvolution from the effects of local tissue macromolecular composition.

## 1 Introduction

An acidic extracellular pH (pH_e_) is a characteristic feature of the tumor microenvironment, with pH_e_ values ranging from 6.2 to 7.4 and falling as low as 3.4–5.5 in some cases [1,2]. This acidic pH is due to lactate production from Warburg metabolism [3] and CO_2_ excretion, due to high catabolic rates and upregulation of the pentose phosphate pathway [1,4]. Larger tumors, such as gliomas, may demonstrate a spatial pH gradient, with a normal pH in the well-perfused periphery and a more acidic pH more centrally [2]. Extracellular acidosis can activate proteinases as well as proangiogenic factors, such as vascular endothelium growth factor A (VEGF-A) and interleukin 8 (IL-8) [5,6], which play a role in stimulating invasion and metastasis [7,8]. pH_e_ may also modulate chemosensitivity and radiosensitivity through alterations in tissue ion trapping [9,10]. Additionally, intracellular pH has been described as a possible marker for delineating salvageable tissue after ischemic stroke with greater accuracy, thus aiding physicians in making treatment decisions [11]. The non-invasive imaging of pH_e_ could therefore be a powerful tool for tumor diagnosis and monitoring of treatment response.

A number of approaches have been used with magnetic resonance imaging (MRI) to non-invasively measure pH, including ^1^H, ^31^P, and ^19^F MR spectroscopy (MRS), hyperpolarized ^13^C MR spectroscopic imaging, chemical exchange saturation transfer (CEST) methods, including endogenous CEST MRI such as amide proton transfer MRI and amide concentration-independent detection (AACID), and techniques like acidoCEST, which employ exogenous agents [[12], [13], [14], [15], [16]]. *R*_1ρ_ MRI is another MRI approach which has been used to measure pH [17]. The *R*_1ρ_ signal, the reciprocal of *T*_*1*_ relaxation in the rotating frame (*T*_1ρ_), can be partially attributed to the exchange of protons between water and proteins. This exchange is pH-dependent and can theoretically be used to image pH at high spatial resolution [17,18]. Given the sensitivity of this method has been reported in the pH range of 6–8, which covers physiological and tumor pH, it could be an attractive technique for non-invasive pH monitoring *in vivo* [19]. Recent work has suggested that *R*_*1ρ*_ measurements are sensitive enough to detect changes in pH in both the murine and human brains following systemic alterations in pH and neuronal activation within the visual cortex [19].

Tissue pH in the brain is modulated by a wide variety of physiological factors, including lactate and CO_2_ production [20], HCO_3_^–^ transport [21], and ionic alterations during neurotransmission [22]. These pH changes may be accompanied by local microenvironmental effects such as vasodilatation and transmembrane ion transport [23,24]. Any method that measures tissue pH must be independent of these changes to accurately measure pH. Here, we investigate the sensitivity of *R*_1ρ_ to pH and macromolecular density, both *in vitro* and in human volunteers, to determine whether the reported pH-dependence of *R*_1ρ_ is independent of these factors.

## 2 Methods

### 2.1 *R*_*1ρ*_ sequence

**Fig. 1:**
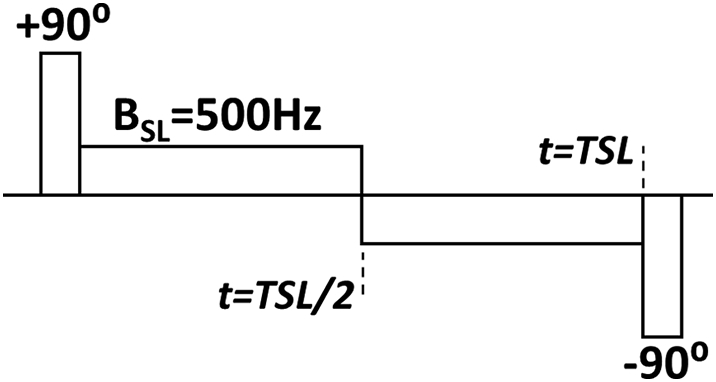
Sequence diagram. The *R*_1ρ_ preparation shown here includes a + 90° tip down pulse, a spin-lock pulse for the length of time TSL (with a 180°phase shift in the centre of the pulse), and a − 90° tip up pulse.

A 3D Fast Spin Echo (3D-FSE) sequence modified to incorporate a spin-lock [25,26] was used on phantoms and humans to measure *R*_1ρ_ relaxation using a 12-channel head coil at 3 T (MR750, GE Healthcare, Waukesha, WI, US). The spin-lock preparation pulse used was hard pulse (90°) - spin-lock (B_1,SL_ = 500 Hz) - hard pulse (−90°), with a (180°) phase transition at the mid-point of the spin-lock pulse. The 180° phase transition was used to reduce B_1_ artefact [15]. The imaging parameters were as follows: spin-lock times (TSL) = 1, 2, 15, 25, and 40 ms; TR = 1587 ms; resolution 0.3 × 0.3 mm^2^; matrix 320 × 256; an echo train length (ETL) of 45; pa Fourier sampling (NEX = 0.5); coil acceleration (ASSET) of 2 [27]. *R*_1ρ_ maps were calculated using linear least-squares regression. A sequence diagram has been provided (Fig. 1).

### 2.2 Phantom preparation

Phantoms of varying pH were made using agarose, milk, bovine serum albumin (BSA), and gadolinium-containing contrast agent (GdCA) (Gadovist, Bayer, Berlin). pH was adjusted by titrating high performance capillary electrophoresis (HPCE) buffer solutions at pH 6.5 and 8.5 (20 mM Na_2_PO_4_), NaOH/HCl, and monosodium phosphate and disodium phosphate solutions (100 mM Na^+^), as shown in Table 1. To control for any effect of sodium concentration on proton exchange, the phantoms had a fixed sodium concentration before pH adjustment with HCl and NaOH (Table 1). To assess the effect of the HPCE buffer on proton exchange, phosphate-buffered (PBS) and aqueous (HCl/NaOH) GdCA-doped phantoms without HPCE were also used. pH was determined using an automatically calibrated bench top electrode pH meter.

**Table 1:** Summary table detailing phantom compositions. BSA = Bovine serum albumin. PBS = Phosphate Buffer Solution. Ag = Agarose.

Phantom	Composition
Fig. 2	GdCA (Gadovist®) concentrations of 0.00 mM, 0.36 mM, 0.72 mM and 1.44 mM in HPCE buffer solution (20 mM Na_2_PO_4_) titrated to pH 6.5, 7, 7.5 or 8.
Fig. 3 ‘PB’	Phantom indicated as ‘HCl’ was made up of sodium phosphate monobasic monohydrate 0.1 M (0.1 M Na+) and GdCA (Gadovist®) (0.72 mM), at pH 4.5. Phantom indicated as ‘NaOH’ was made up of sodium phosphate dibasic heptahydrate 0.05 M (0.1 M Na+) and GdCA (Gadovist®) (0.72 mM), at pH 9.0 For both phantoms, pH was adjusted to 4.5 or 9.0 with HCl and NaOH.
Fig. 3 ‘PB/BSA’	Phantom indicated as ‘HCl’ was made up of sodium phosphate monobasic monohydrate 0.1 M (0.1 M Na+) and BSA 8% wt/v, at pH 4.5 Phantom indicated as ‘NaOH’ was made up of sodium phosphate dibasic heptahydrate 0.05 M (0.1 M Na+) and BSA 8% wt/v), at pH 9.0 For both phantoms, pH was adjusted to 4.5 or 9.0 with HCl and NaOH.
Fig. 3 ‘Gad (aq)’	GdCA (Gadovist®) concentration of 0.72 mM in aqueous solution. For both phantoms, pH was adjusted to 4.5 or 9.0 with HCl and NaOH.
Fig. 3 ‘PBS/BSA/Ag’	Phantom indicated as ‘HCl’ was made up of sodium phosphate monobasic monohydrate 0.1 M (0.1 M Na+), BSA 8% wt/vol and 3% agarose wt/vol, at pH 4.5 Phantom indicated as ‘NaOH’ was made up of sodium phosphate dibasic heptahydrate 0.05 M (0.1 M Na+), BSA 8% wt/vol and 3% agarose wt/vol, at pH 9.0 For both phantoms, pH was adjusted to 4.5 or 9.0 with HCl and NaOH.
Fig. 4 ‘BSA’	All phantoms prepared with GdCA (Gadovist®) (0.72 mM). BSA concentration was adjusted to 0%, 0.1875%, 0.375%, 0.5%, 0.75%, 1%, 1.5%, 2%, 3%, 4%, 6% and 8% wt/vol Phosphate buffer used was prepared from mixture of sodium phosphate monobasic monohydrate 0.1 M (0.1 M Na+) and sodium phosphate dibasic heptahydrate 0.05 M (0.1 M Na+) to pH 7.20.
Fig. 4 ‘Milk’	All phantoms prepared with GdCA (Gadovist®) (0.72 mM). Nido Full Cream Milk Powder (Nestlé ©) was used to adjust milk concentration to 0%, 0.625%, 0.9375%, 1.25%, 1.875%, 2.5%, 3.75%, 5% and 7.5% wt/vol Phosphate buffer used was prepared from mixture of sodium phosphate monobasic monohydrate 0.1 M (0.1 M Na+) and sodium phosphate dibasic heptahydrate 0.05 M (0.1 M Na+) to pH 7.20.

**Fig. 2:**
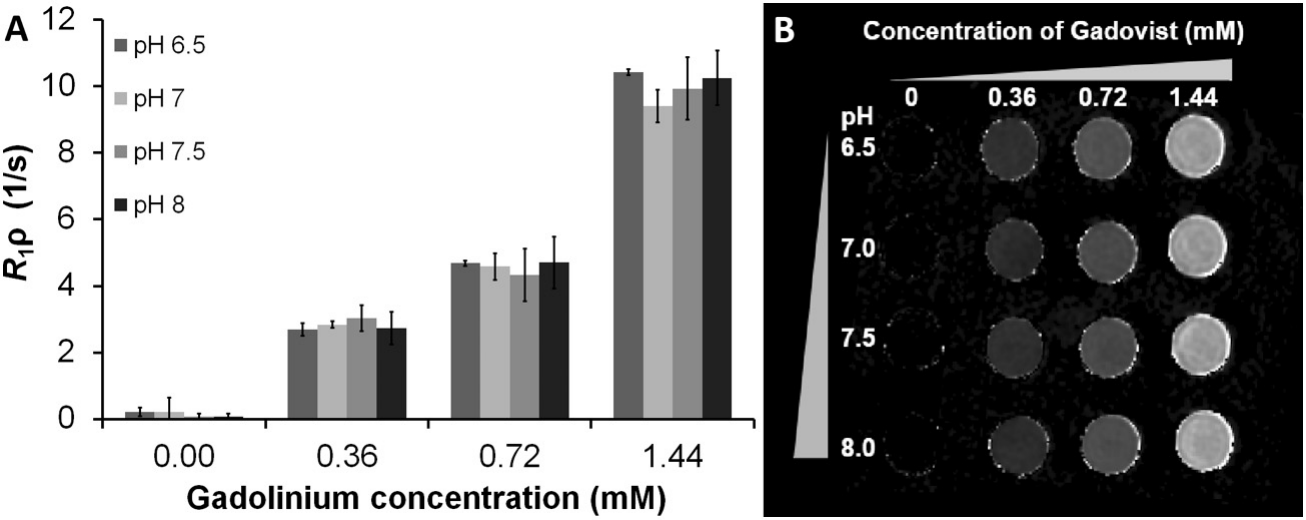
Sensitivity of *R*_1ρ_ to pH and GdCA chelate concentration. (A) *R*_1ρ_ measurements derived from *R*_1ρ_ maps of HPCE-buffered phantoms in the pH range 6.5–8.0 with varying GdCA concentration (mean ± standard deviation). *R*_1ρ_ was shown to vary with GdCA concentration but not pH in the absence of macromolecules. (B) Representative image of phantoms from Fig. 2A scanned with R_1ρ_.

A gadolinium containing contrast agent (GdCA) was added to increase the *T*_1ρ_ relaxation rate, as the relaxation time without GdCA was too long (>200 ms) to measure with the given spin-lock times reliably, without increasing signal non-uniformity. The concentration of GdCA added (0.36 mM) was calculated to approximate the extracellular fluid (ECF) concentration achieved when 5 mL of 1.0 M GdCA is injected during an *in vivo* study, assuming an average ECF volume of 14 L in a 75 kg male adult [28]. It was determined from the results of varying GdCA concentration in Fig. 2 that a concentration of 0.72 mM GdCA produced an optimal range of *T*_1ρ_ relaxation rates, and this concentration was used in subsequent experiments.

To study the effect of protein and macromolecular concentration on the pH-dependence of *R*_1ρ_, phantoms with a fixed pH, but varying concentrations of BSA or milk powder, were made up to the maximum saturation level that could be achieved. Previous studies have used glutaraldehyde or agarose but as these produced solid or gelatinous phantoms, the pH could not be reliably measured after setting [17,19]. To maintain the ability to monitor pH throughout the phantom-making process, phantoms were first made without the addition of glutaraldehyde or agarose, as have been used previously [19]. Agarose was subsequently added to examine the effect of macromolecular density.

All phantom samples were scanned together inside a single water bath, such that any heating or cooling that occurred affected all samples together. The B_1,SL_ transmit power was low due to clinical system constraints, which are in place to prevent heating. The temperature of the water bath measured with an infrared thermometer (ST-8861, aml Instruments, Lincoln, UK) before and after a *R*_1ρ_ experiment had <0.5 °C difference.

### 2.3 Phantom *R*_1ρ_ analysis

Long spin-lock preparation of *R*_1ρ_ may introduce artefacts related to B_0_ and B_1_ inhomogeneities. To reduce the effects of artefacts on *R*_1ρ_ values, we randomized the sample position in the field of view between scans and averaged the *R*_1ρ_ values across three acquisitions to obtain a single *R*_1ρ_ value per sample. This randomization procedure may not fully account for non-linear B_0_ and B_1_ non-uniformity across the sample, but it does represent an average of the field non-uniformities and minimizes effects from variations in B_0_ and B_1_. Phantoms were imaged while submerged in water before *R*_*1ρ*_ imaging to reduce susceptibility effects from surrounding air.

A linear least-squares analysis was performed to calculate Pearson correlation coefficients and statistical significance between *R*_1ρ_ measurements and the pH of the phantom or macromolecular concentration.

### 2.4 Human *R*_1ρ_ imaging and analysis

*R*_1ρ_ images were acquired from 7 healthy male volunteer brains (median age 21, range 20–22). As there are known variations in macromolecular density between white and grey matter in the brain [29], we assessed regional variations in *R*_1ρ_. The ROIs were positioned in manually matched anatomical regions by a single operator, defined as white matter (frontal lobe, parietal lobe, occipital lobe, internal capsule, corpus callosum, centrum semiovale), grey matter (frontal lobe, parietal lobe, occipital lobe), and basal ganglia (putamen, caudate). ROIs of approximately equal size were manually placed in 10 different positions bilaterally within each matched anatomical region for each volunteer, and their values averaged. Normal distribution was tested with a quantile-quantile (Q-Q) plot and a two-tailed *t*-test was used to calculate statistical significance for *R*_1ρ_ differences between white and grey matter, white matter and basal ganglia, and grey matter and basal ganglia.

## 3 Results

**Fig. 3:**
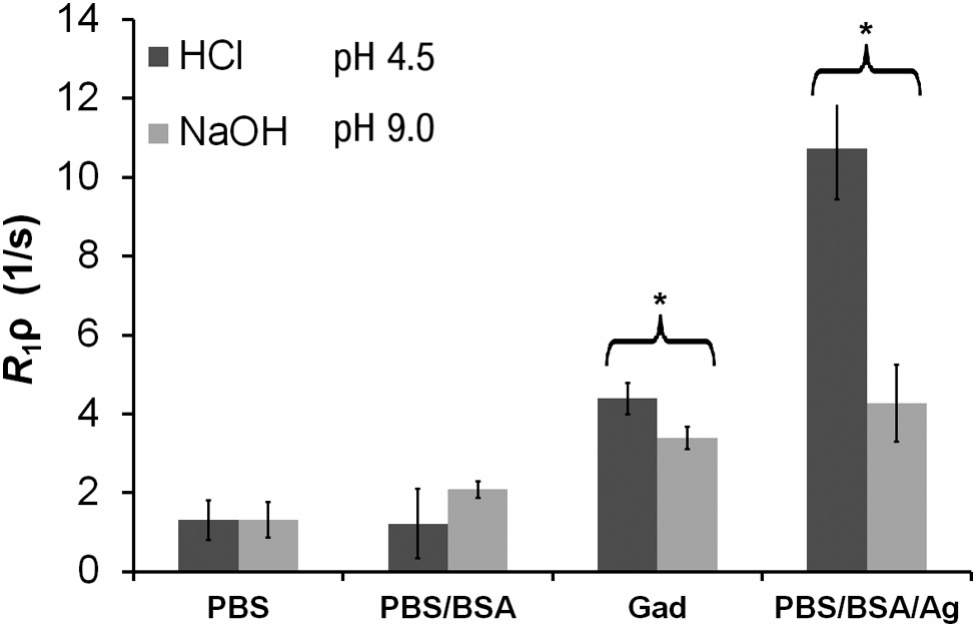
Sensitivity of *R*_1ρ_ to pH and phantom composition. *R*_1ρ_ measurements derived from maps of phantoms of pH 4.5 and 9 prepared with phosphate buffer saline (PBS, 0.1 M Na+), PBS and bovine serum albumin (PBS/BSA; 0.1 M Na+, BSA 8% wt/v), aqueous GdCA (0.72 mM) (Gad), and PBS, BSA and agarose (PBS/BSA/Ag; 0.1 M Na+, BSA 8% wt/v, 3% agarose wt/vol). *p < 0.05 significance in *R*_1ρ_ signal.

The phantom experimental design was used to assess the correlation between *R*_1ρ_, pH and the concentration of macromolecules and proteins. We observed no significant difference in *R*_1ρ_ between HPCE-buffered phantoms at pH 6.5, 7.0, 7.5, and 8.0 (Fig. 2). In these phantoms, varying GdCA concentration from 0 to 1.44 mM linearly increased *R*_1ρ_ rates from 0 s^−1^ to 10 s^−1^. In addition, no significant differences in *R*_1ρ_ were observed at the extremes of pH in phosphate-buffered phantoms with or without bovine serum albumin (BSA; pH 4.5 and 9.0; Fig. 3).

In GdCA-doped aqueous phantoms, there was a small, but significant (*p* < 0.05) difference in *R*_1ρ_ measured in phantoms at pH 4.5 and 9.0 (Fig. 3). Unlike the case with BSA, we demonstrated a significant pH-dependence in *R*_1ρ_ when agarose was included in the phantoms (p < 0.05; Fig. 3). In both GdCA-doped aqueous phantoms and agarose-containing phantoms (Fig. 3), there was a dependence of *R*_1ρ_ on pH.

**Fig. 4:**
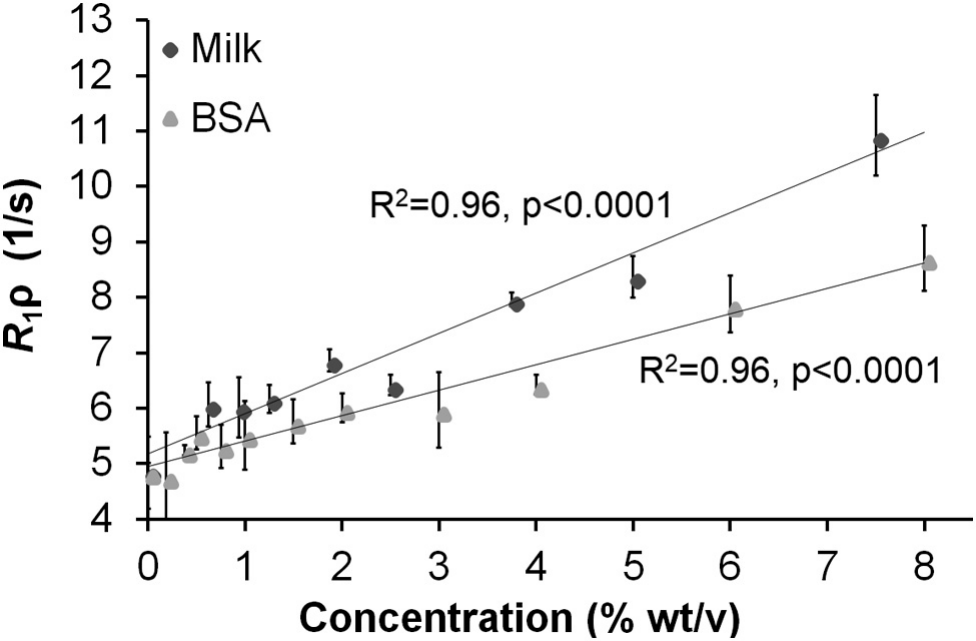
Sensitivity of *R*_1ρ_ to macromolecular concentration. *R*_1ρ_ measurements derived from *R*_1ρ_ maps of phantoms at a fixed pH of 7.2 and varying concentrations of milk (circles) and BSA (triangles). Pearson R score for the milk solution: R^2^ = 0.96, *p* < 0.0001, *R*_1ρ_ = 0.72[milk] + 5.19; Pearson R score for the BSA solution: R^2^ = 0.96, p < 0.0001, *R*_1ρ_ = 0.46[BSA] + 4.96.

*R*_1ρ_ demonstrated the highest degree of sensitivity to pH change in agarose-containing phantoms. pH change was accompanied by visible structural change at low pH, akin to curdling. This prompted us to question whether structural changes that occur as macromolecular concentration changes would also influence *R*_1ρ_. Therefore, we also investigated whether the *R*_1ρ_ signal is dependent on macromolecular concentration, without changes in pH or other variables, which could explain the effects of pH in the presence of agarose. We observed that at constant pH, increasing the phantom macromolecular density with milk powder or BSA from 0 to 8% wt/v correlated with *R*_1ρ_ signal (correlation coefficient 0.96 for both; Fig. 4).

**Fig. 5:**
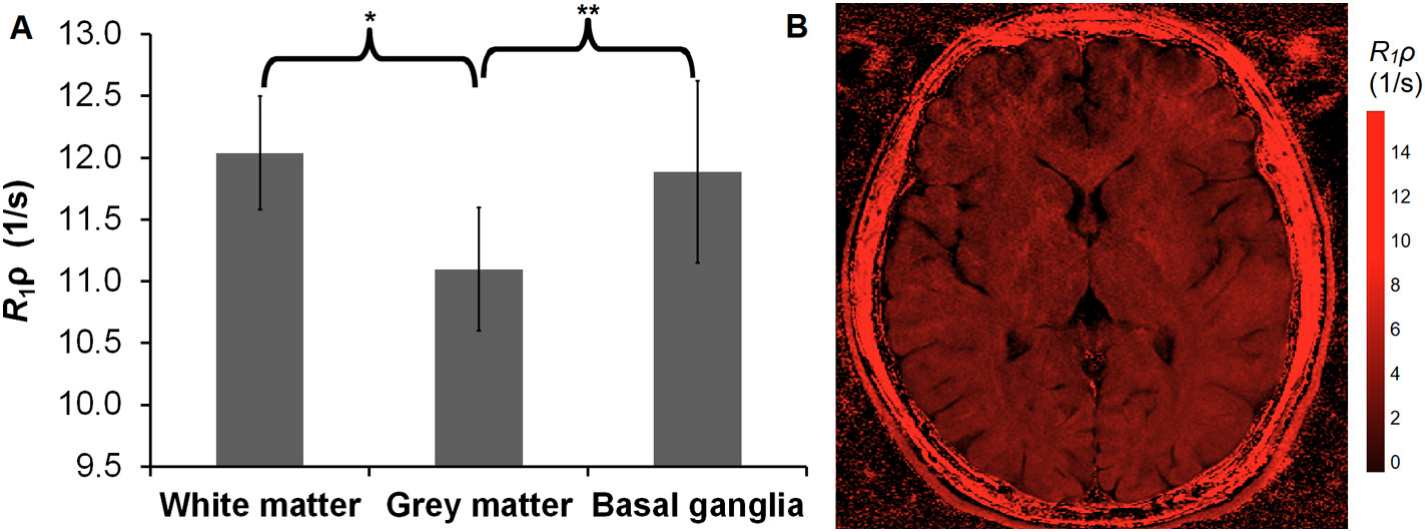
R_1ρ_ data from the human brain. (A) Calculated mean *R*_1ρ_ values (+/− standard deviation) for white matter, grey matter, and basal ganglia in 7 healthy human volunteers (median age 21). Manual ROIs were matched to anatomical regions defined as white matter (frontal lobe, parietal lobe, occipital lobe, internal capsule, corpus callosum, centrum semiovale), grey matter (frontal lobe, parietal lobe, occipital lobe), and basal ganglia (putamen, caudate. ROIs were placed in 10 different positions within each matched anatomical region in each volunteer, and then averaged. Normal distribution was tested with a quantile-quantile (Q-Q) plot and a two-tailed *t*-test was used to calculate statistical significance for *R*_1ρ_ differences between white and grey matter, white matter and basal ganglia, and grey matter and basal ganglia. *p* < 0.001 ** *p* < 0.05; significance in *R*_1ρ_ signal difference. (B) *R*_1ρ_ map of the brain of a healthy volunteer.

*R*_1ρ_ differences were observed between brain regions where there are known differences in macromolecular density [29], with representative imaging shown in Fig. 5B. *R*_1ρ_ imaging demonstrated significant differences between white (12.04 ± 0.46 s^−1^; mean ± SD) and grey matter (11.10 ± 0.50 s^−1^; *p* < 0.001), and between grey matter and basal ganglia 11.89 ± 0.74 s^−1^; *p* < 0.05; Fig. 5A). Therefore, *R*_1ρ_ appears to be sensitive to regional variations in tissue structure throughout the brain which is likely to represent changes in macromolecular concentration rather than changes in pH.

## 4 Discussion

Several methods have been described previously to image the spatial distribution of pH in the brain, including ^1^H, ^31^P and ^19^F spectroscopy, amide proton transfer and hyperpolarized ^13^C MR spectroscopic imaging [17,18,30,31]. However, their use in clinical practice has been limited by lengthy acquisition times, the need for specialized hardware, and poor spatial resolution.

A previous study has reported that *R*_1ρ_ can detect alterations in cerebral pH with changes in inspired gases and following neuronal activation in the visual cortex [19]. MRI contrast relies heavily on the interaction between the nuclear magnetic relaxation of water and macromolecules such as proteins. *R*_1ρ_ has been shown to rely on proton exchange between free water and protein side chain groups in solution [17]. pH affects the water-protein interaction by directly altering protein surface charges and the strength of water-protein hydrogen bonds which in turn alter the exposure of NMR-visible side chains and the dynamics of protein tumbling through tertiary structure changes [17]. It has been shown that any pH-dependence of *R*_1ρ_ is derived from this water-protein proton exchange [17].

However, contrary to the previous published work outlined above, in the absence of these proteins we found no evidence to support that *R*_1ρ_ is sensitive to changes in pH in the range 6.5–8 (Fig. 2), and only shows a small degree of change between the extremes of 4.5 and 9 (Fig. 3), in GdCA-containing phantoms. Indeed, we found that the sensitivity of *R*_1ρ_ to physiological pH changes in GdCA-containing phantoms, in the absence of macromolecules, was much lower than that previously reported [19]. However, we have demonstrated that *R*_1ρ_ measurements are highly sensitive to changes in pH in the presence of agarose, where the pH change is accompanied by visible structural alterations, but not in the presence of albumin without agarose, where such pronounced structural alterations do not occur (Fig. 3). The idea that changes in structural density affect *R*_1ρ_ signal was further tested with measurements from phantoms composed of different concentrations and types of macromolecules: milk as a mixture of lipids, proteins and carbohydrate, and BSA as a homogeneous protein solution. These all showed a correlation of *R*_1ρ_ signal with macromolecular density (Fig. 4).

pH results in relaxation rate changes due to its modification of free H^+^ or OH^–^ molecules and decrease in the activation energy required for proton spin exchange (by 14% between pH 6 and 8) [32]. A flat dependence of *R*_1_ and *R*_2_ for pH values between 6 and 8 was reported by others [33], which is related to *R*_1ρ_ since all three parameters share a dependence on “molecular correlation time” [34]. In PBS/BSA/agarose phantoms, the correlation between *R*_1ρ_ and pH observed in our work was the reverse of that previously reported for similar phantoms, *i.e.* decreasing *R*_1ρ_ with increasing pH here compared to increasing *R*_1ρ_ with increasing pH previously [19]. We were able to replicate the experiments of [19] when using the agarose/PBS/BSA phantom (Fig. 3), where pH related changes would cause structural alteration in the phantom and induce other relaxation effects.

Overall, *R*_1ρ_ with a spin-lock power of 500 Hz can only be used as a biomarker of pH when corrected for regional macromolecular density, which is not a trivial measurement. Our findings indicate that *R*_1ρ_ is an unreliable measure of dynamic pH changes in functional imaging, or in tumor monitoring, where the tissue architecture changes as the tumor grows. These experiments showed *R*_1ρ_ increased linearly with increasing macromolecular density at a constant physiological pH of 7.20 (Fig. 4). Previous work has shown that cross-linking BSA with glutaraldehyde to limit the number of NMR-visible side chain groups attenuates the sensitivity of *R*_1ρ_ to the spin-lock field but did not test the relationship between pH sensitivity and macromolecule concentration [17]. Here we have shown that the macromolecular concentration over the range 0.00–8.00% wt/v correlates with *R*_1ρ_ signal at fixed pH. Proteins and other macromolecules may vary considerably in concentration across normal tissues as well as in the heterogeneous tumor microenvironment [2]. In addition, while the relaxation rate linearly correlates with macromolecular concentration and type over the range tested, non-linear effects are possible at higher concentrations after chemical saturation. Interestingly, similar conclusions have been drawn in studies of pH imaging with the endogenous CEST MRI method, amide proton transfer (APT) MRI. There, the contrast-enhancing effects of increased protein content within tumors may oppose the contrast-reducing effect of lower tumor pH producing only a small increase in APT contrast of the tumor compared to the surrounding tissue [35].

One of the limitations of this experiment is how to disentangle the different elements that contribute to *R*_1ρ_ relaxation. The difficulty of the experiment is due to the measured *R*_1ρ_ being affected by the *R*_1ρ_ of each chemical component within the mixture, such that the pH-dependent portion of *R*_1ρ_,_exchange_ cannot be disentangled as long as it remains a smaller effect than the relaxation rate changes induced by the macromolecular and buffer components, which are necessary to create an altered pH state. A simple model of relaxation rate consists of a linear sum from multiple chemical components, such that our expected *R*_1ρ_ would be:

$$R_{\text{1ρ}}=R_{\text{1ρ},H20}+R_{\text{1ρ},\text{exchange}}+R_{\text{1ρ},\text{macromolecule}}+R_{\text{1ρ},\text{buffer}}+R_{\text{1ρ},\text{field}}+R_{\text{1ρ},\text{other}}$$

where the pH dependence of the equation results from the chemical exchange (*R*_1ρ_,_exchange_) between the free water and labile protons [33]. Non-uniform B_0_ and B_1_ contribute to signal decay through the *R*_1ρ,field_ term. *R*_1ρ,other_ accounts for any impurities [32]. While a more complicated model is required to more completely describe the molecular concentrations and interdependence of the terms, this model is useful for first order analysis and conceptual understanding. The buffer required to modify the pH, which has both buffer and pH-related relaxation effects, creates a significant confounder in determining pH-related changes and has been raised as a concern in other studies [32].

More complicated models exist, such as methods that attempt to estimate exchange kinetics [32] and models that remove the relaxation portions due to diffusion or where random molecular motion lead to *R*_1ρ,H20_ being equal to *R*_2,H20_ and *R*_1,H2O_ [36], which require spin-lock fields higher than 3000 Hz [[36], [37], [38], [39]] and cannot be achieved on our clinical system due to patient safety constraints. It may be possible to use much smaller spin-lock fields to measure pH [32], where *R*_1ρ_ is nearly indistinguishable from *R*_2_, however such measurements in clinical settings would be hampered by increased field non-uniformity effects that confound low spin-lock *R*_1ρ_ measurements. Our study is unique in using variations of pH buffers for investigating a moderate (500 Hz) spin-lock field [32].

In addition to multiple chemical components contributing to *R*_1ρ_ relaxation, B_0_ and B_1_+ can complicate experimental design. We designed our experiment to minimize B_0_ and B_1_+ non-uniformities that will increase the phantom relaxation rate. Due to the use of the standard radiofrequency body coil, and a relatively small phantom, we expected that field non-uniformities would arise primarily at the boundaries of the phantom and air, and therefore we maximized the distance between any pH phantom and the sides of the larger water-containing phantom. While B_0_ and B_1_+ non-uniformities are not visible in Fig. 2, we considered even small B_0_ and B_1_+ effects, as well as a low signal-to-noise ratio, as potential confounders. To counteract these effects, we surrounded our phantoms with water to reduce B_0_ inhomogeneity that occurs on the boundaries between water and air, performed gradient shimming, and used positional randomization under the assumption that any non-uniformities that existed did not occur over the entire physical volume.

We did not see significant non-uniformity in the *R*_1ρ_ maps, but consider B_0_ and B_1_ non-uniformity as contributors to the errors visible on Fig. 1, Fig. 2, Fig. 3, which was smaller than the change visible on most buffer measurements. A significant increase in signal non-uniformity can be seen for spin-lock times longer than used here, which is subject to both the transmit B_1_^+^ and the static B_0_ fields. We used a phase transition in the spin-lock pulse to reduce B_1_+ effects, although methods that would reduce these artefacts further include the use of adiabatic 90° pulses or a central 180°pulse [40]. A greater spin-lock B_1_ (B_1,SL_) pulse would be less subject to B_0_ non-uniformity [40], however, the maximum B_1,SL_ power is limited on this clinical system to ensure patient safety, and B_1,SL_ of 500 Hz is commonly used for *in vivo* experiments, and B_1,SL_ = 400 Hz has previously been used to show a pH dependence [19]. A 1.2 kHz, 135° pulse prior to the spin-locking could reduce the B_0_ sensitivity, however, the power required to achieve is not considered practical [40]. Slightly longer spin-lock times are sometimes used (up to 100 ms) in other T_1ρ_ studies [41], although these times increase signal non-uniformities, limiting the accuracy of T_1ρ_ relaxation time measurements above ~150 ms (*R*_1ρ_ < 6 s^−1^). Adiabatic 90° pulses can be used to reduce field non-uniformity artefact, however, we used positional randomization to address artefacts. As the 90° pulses were unaltered with several spin-lock times, the system should be dependent primarily on the spin-lock times and thereby *R*_1ρ_.

While pH changes with GdCA may not perfectly match tissue environments, a correlation between pH and *R*_1ρ_ should have been seen if *R*_1ρ_ is to be considered relevant for spin-lock times <100 ms. This study did not assess whether *R*_1ρ_ would have a stronger relationship with pH when measured with longer spin-lock times, however, long spin-lock times are not used when *R*_1ρ_ and pH is referenced being correlated [[42], [43], [44]]. Although pH may directly affect *R*_1ρ_, it remains a small effect without the presence of other macromolecule effects.

We investigated regional variations in *R*_1ρ_ across the brain and found significant differences in signal between structurally distinct brain regions. White matter and basal ganglia regions had a higher *R*_1ρ_ compared to grey matter (*p* < 0.001) which is likely to be accounted for by the higher content of both protein and lipid [29]. Importantly, no significant differences in intracellular pH between grey and white matter have previously been found using ^31^P NMR techniques [45]. Measurements of pH in the brain using *R*_1ρ_ are likely to be affected by the concentration of hyaluronic acid and chondroitin sulphate. Regional activity-dependent variations in blood flow have been shown to affect *R*_1ρ_ [19], as well as cerebral ischemia [46,47]; increases in blood volume may alter macromolecular concentrations, which in turn could affect *R*_1ρ_ independently of pH. Future studies are required to more fully quantify the effect of macromolecular concentration and blood flow on *R*_1ρ_ before it can be used as an *in vivo* marker of pH.

In conclusion, we have demonstrated that the pH-dependence of *R*_1ρ_ MRI is highly sensitive to changes in macromolecular concentration and that it is unreliable as a measure of pH alone without taking these factors into consideration. We have also demonstrated variations in *R*_1ρ_ across the normal human brain. These findings have important implications for its applications to studying activity-dependent neuronal activation, as well as its potential role in tumor imaging.
